# Beneficial effects of silver foam dressing on healing of wounds with ulcers and infection control of burn patients

**DOI:** 10.12669/pjms.316.7734

**Published:** 2015

**Authors:** Bo Yang, Xudong Wang, Zhonghua Li, Qi Qu, Yan Qiu

**Affiliations:** 1 Bo Yang, Department of Burn and Plastic Surgery, The Fourth People’s Hospital of Jinan City, Jinan 250031, China; 2 Xudong Wang, Department of Burn and Plastic Surgery, Zaozhuang Municipal Hospital, Zaozhuang 277102, China; 3 Zhonghua Li, Department of Burn and Plastic Surgery, The Fourth People’s Hospital of Jinan City, Jinan 250031, China; 4 Qi Qu, Department of Burn and Plastic Surgery, The Fourth People’s Hospital of Jinan City, Jinan 250031, China; 5 Yan Qiu, Department of Hematology, Qilu Hospital of Shandong University, Jinan 250031, China

**Keywords:** Silver foam dressing, Second-degree burn, Wound infection, Ulcer

## Abstract

**Objective:**

To assess the beneficial effects of silver foam dressing on the healing of wounds with ulcers and infection control of burn patients.

**Methods:**

Eighty-four second-degree burn patients were selected and divided into a study group and a control group (n=42). After disinfection and cleaning, wound beds of the study group were covered with silver-containing soft-silicone foam dressing, and wound surfaces of the control group were wiped with 1% silver sulfadiazine cream (60 g/100 cm^2^). The two groups were checked weekly to observe wound healing progress and adverse reactions of the skin around wounds. Wound secretions were collected and subjected to bacterial culture. Related indices were recorded and quantified.

**Results:**

Thirty seven cases of the study group (88.1%) and 36 cases of the control group (85.7%) recovered to normal, and 3 (7.1%) and 2 cases (4.8%) in the two groups failed to recover. The recovery rates of the two groups were similar (P>0.05), but unrecovered patients in the study group had significantly higher proportions of repaired wounds (P<0.05). Wounds of the study group were healed significantly more rapidly than those of the control group (22.3±3.1 vs. 25.1±4.4, P<0.05). The study group had significantly higher proportions of repaired wounds from Day 7 to Day 21 (P<0.05), but the difference became less obvious with extended time to Day 28. The bacterial culture-positive (exceeding 10^5^ organisms per gram of tissue) rates of both groups significantly reduced after treatment (Day 7 for the study group and Day 14 for the control group), and the rate of the study group was significantly lower at last (P<0.05). The study and control groups were observed 134 and 149 person-times respectively, with the normal wound-surrounding skin rates of 96.3% (129/134) and 88.6% (132/149) (P>0.05 except for on Day 14). Except for on Day 28, the study group had significantly lower pain scores than those of the control group (P<0.05), especially on Day 7 and Day 14 (P<0.01). From Day 7 to Day 28, the study group was significantly less prone to burning sensation than the control group (P<0.05), but both groups felt anxious during dressing change (P>0.05). Dressing of the study group was changed significantly more easily (P<0.05), but the fixing outcomes were similar (P>0.05).

**Conclusion:**

Silver foam dressing rapidly, easily and safely resisted wound bacteria, promoted wound healing and shortened recovery time, effectively relieving the pain of patients.

## INTRODUCTION

Burn has now ranked the 4th most common cause of injury right after traffic accident, tumbling and violence, severely threatening global public health. Particularly, wounds of second-degree burn are painful and may form scars and ulcers upon aggravation, which lead to disfigurement or partial loss of function and thus seriously affect the quality of life.^1^-^3^ The wounds from which ulcers arise can be caused by various factors, including bacterial or viral infections, fungal infections, radiation and drugs. Ulcers of burn wounds are commonly induced by infections.^4^-^6^ Infections severely interfere with wound healing because bacteria vigorously proliferate to defeat leukocytes and thus result in redness and swelling, impregnation and exudates. Besides rational nutritional support and use of antibiotics, proper dressing is also needed to control local infections.

Proper dressings should cover wound surfaces, resist bacterial invasion, prevent infections and most importantly, participate in the process of wound healing, without toxicity or adverse reactions.^7^-^9^ Burn wounds are often treated with 1% silver sulfadiazine which, however, stops functioning due to increased bacterial drug resistance. Meanwhile, silver sulfadiazine leads to pain and even wound aggravation.^10^,^11^ In contrast, silver foam dressing, which combines inorganic silver and dressing, is broad-spectrum, long-lasting and can promote burn wound healing effectively without obvious drug resistance.

In this study, we compared the effects of these two dressings on the healing of wounds with ulcers and infection control of burn patients.

## METHODS

### Subjects

Eighty-four burn patients treated in our hospital from June 2010 to September 2014 were selected. This study was been approved by the ethics committee of Qilu Hospital of Shandong University, and written consent has been obtained from all patients.

### Inclusion criteria

Females or males aged 18-60 years old; with second-degree burns (deep, superficial) on non-joint regions; total burn area< 25% of total body surface area (TBSA); with wound infections or ulcers.

### Exclusion criteria

With burn injuries caused by chemical substances, electric shock or radiation; with heart, liver or renal insufficiency; with severe dystrophy; with skin diseases or others affecting treatment (e.g. HIV/AIDS, cancer and severe anemia); allergic or hypersensitive to experimental materials; with burns on the head, face, perineum or joints; pregnant women or patients with mental diseases and failed to comply with treatment.

### Positions for study

Individual burn regions with the areas of 1%-5% TBSA were selected (special positions excluded).

The 84 eligible patients comprised 49 males and 35 females. All wounds suffered from rupture and ulceration, and sloughs on the wound bed were accompanied by yellow pus, local redness and swelling and even unpleasant smell. They were then divided into a study group and a control group (n=42), and their age, gender composition, wound area, area for study and causes of burn were similar (Table-I).

**Table-I:** Baseline clinical data.

	Study group	Control group	P	Total
Gender (male/female)	26/16	23/19	>0.05	49/35
Age (year)	34.9±10.2	36.2±11.4	>0.05	35.6±10.7
Infection time (h)	10.5±2.8	9.3±3.3	>0.05	9.8±2.9
Burn area (%TBSA)	9.7±4.1	9.2±4.9	>0.05	9.5±4.4
Area for study (%TBSA)	3.0±0.6	2.9±0.9	>0.05	3.0±0.8
*Cause of burn*				
Fire	18 (42.9%)	20 (47.6%)	>0.05	38
Hot fluid	16 (38.1%)	15 (35.7%)	>0.05	31
Thermal contact	8 (19.0%)	7 (16.7%)	>0.05	15

### Methods

### Wound treatment

Wounds were first sterilized inwards by using 0.05% chlorhexidine acetate (Xibitai) and debrided based on the physical status of patients. For the patients with strong resistance or the young ones, mechanical debridement was performed under local anesthesia with pliers, tweezers and scissors to remove all narcotic tissues such as sloughs and pus. As to the elderly or patients with poor physical state, autolytic debridement was conducted, i.e. necrotic tissues were lysed and removed depending on naturally produced proteolytic enzymes. After debridement, wound beds were cleaned with hydrogen peroxide and then with a large amount of normal saline.

### Wound treatment

For the study group, silver-containing soft-silicone foam dressing (ChinaGate Company Ltd., batch No. 10-3361) tailored to 2-5 cm larger than the wound was covered, fixed and protected with a secondary dressing. The silver foam dressing should be closely adherent to the wound bed, and if not, additional materials such as cotton balls were required to render the dressing to adapt to a depressed or irregularly shaped wound bed. The dressing was changed (once per day to once per week) according to wound healing and exudation outcomes.

Wounds of the control group were wiped with 1% silver sulfadiazine cream (60 g/100 cm^2^, Zhunzi 1998-0481) and bandaged with gauze. The dressing was refreshed once per day or between a shorter time if considerable purulent secretions were exuded. In the meantime, drainage was enhanced.

### Evaluation criteria

The two groups were observed five times for 28 days in total on Day 0, Day 7, Day 14, Day 21 and Day 28 respectively.

### Wound healing

Wound area, degree of recovery and healing time were recorded. To circumvent subjective errors, wounds were photographed and analyzed by using Image Pro-plus software to calculate the proportion of repaired wound.

Proportion of repaired wound = (Initial area of wound - area of unhealed wound)/initial area of wound × 100%.

A wound was considered recovered to normal if the proportion of repaired wound reached 95%.

### Culture of wound bacteria

Wound secretions were cultured to detect the presence of common skin surface bacteria including *Staphylococcus aureus*, *Escherichia coli*, *Staphylococcus epidermidis*, *Pseudomonas aeruginosa* and hemolytic staphylococci.

Detection rate of bacteria = (Number of wounds with positive culture results - total number of wounds) × 100%. Where positive culture results mean the levels of bacteria exceed 10^5^ organisms per gram of tissue.

### Wound-surrounding skins

Whether skins suffered from impregnation, erythema, rash and eczema was observed.

### Assessment of dressings

Pain, anxiety and burning sensation were inquired and recorded every time before wound treatment and upon dressing change, and the difficulty and fixing of changing dressing were compared.

Visual analogue scale was used to assess the degree of pain, with 0 representing painless (100 points in total). Subjective indices including anxiety, burning sensation, as well as difficulty and fixing of changing dressing were quantified with five levels (5 points: excellent; 4 points: good; 3 points: fair; 2 points: poor; 1 point: very poor). For example, 5 points meant no burning sensation.

### Statistical analysis

All data were analyzed by SPSS 15.0 software and first subjected to normality test. The categorical data were expressed as (mean ± standard deviation), and inter-group comparisons were performed with t test. The numerical data were expressed as percentage (%)m and intergroup comparisons were conducted by using χ^2^ test. P<0.05 and P<0.01 were considered as significant difference and markedly significant difference.

## RESULTS

### Treatment outcomes

At the end of this study, 37 cases of the study group (88.1%) and 36 cases of the control group (85.7%) recovered to normal, and 3 (7.1%) and 2 cases (4.8%) in the two groups failed to do so respectively. Besides, 2 (4.8%) and 4 cases (9.5%) in the two groups were surgically treated respectively. The recovery rates of the two groups were similar (P>0.05), but unrecovered patients in the study group had significantly higher proportions of repaired wounds (88%, 90% and 91% vs. 65% and 78%) (P<0.05).

### Wound healing

### Wound healing time

The wound healing times of the study and control groups excluding the unrecovered patients were 18-24 d (average: 22.3±3.1) and 20-28 d (average: 25.1±4.4) respectively. The difference was statistically significant (P<0.05).

### Proportion of repaired wound

The study group had significantly higher proportions of repaired wounds from Day 7 to Day 21 (P<0.05), but the difference became insignificant with elapsed time to Day 28 (Table-II).

**Table-II:** Proportions of repaired wounds.

Proportion of repaired wound	Study group (%)	Control group (%)	P	Total
Day 7	30.8±13.1	14.2±11.9	0.009	21.5±12.3
Day 14	68.1±16.6	47.7±14.5	0.016	57.7±15.0
Day 21	94.3±8.4	86.0±10.3	0.022	90.2±9.6
Day 28	98.1±1.4	96.3±2.0	0.061	97.7±1.7

### Bacterial culture of wound secretions

On Day 21, 33 patients in the study group and 39 in the control group continued to participate in the experiment (others recovered), which became 17 and 26 respectively on Day 28. The bacterial culture-positive (exceeding 10^5^ organisms per gram of tissue) rates of the two groups were 54.8% and 50% respectively initially, which significantly reduced after treatment (Day 7 for the study group and Day 14 for the control group), and the rate of the study group was significantly lower at last (P<0.05) (Table-III).

**Table-III:** Bacterial culture-positive (exceeding 10^5^ organisms per gram of tissue) cases.

Bacterial culture	Study group (case number)		Control group (case number)		P	Total	
	n	Positive	n	Positive		n	Positive
Day 0	42	23 (54.8%)	42	21 (50%)	>0.05	84	44 (52.3%)
Day 7	42	10 (23.8%)^a^	42	17 (40.4%)	0.031	84	27 (32.1%)
Day 14	42	6 (14.3%)^a^	42	15 (35.7%)^b^	0.024	84	21 (25%)
Day 21	33	2 (6.1%)^aa^	39	10 (25.6%)^b^	0.011	72	12 (16.7%)
Day 28	17	1 (5.9%)^aa^	26	4 (15.4%)^bb^	0.016	43	5 (11.6%)

Results of the study group on Day 7-Day 28 compared with those on Day 0,

a *P<*0.05,

aa *P<*0.01; results of the control group on Day 7-Day 28 compared with those on Day 0,

b *P<*0.05,

bb *P<*0.01.

### Wound-surrounding skins

The study and control groups were observed 134 and 149 person-times respectively, with the normal wound-surrounding skin rates of 96.3% (129/134) and 88.6% (132/149) (P>0.05 except for on Day 14) (Table-IV).

**Table-IV:** Wound-surrounding skins.

Wound-surrounding skin	Study group	Control group	P	Total
Day 7	n=42	n=42		n=84
Normal	41 (97.6%)	38 (90.5%)	0.305	79 (94.0%)
Erythema	0	2		2
Impregnation	1	1		2
Rash	0	1		1
Day 14	n=42	n=42		n=84
Normal	42 (100%)	35 (83.3%)	0.029	77 (91.7%)
Erythema	0	3		3
Impregnation	0	3		3
Rash	0	1		1
Day 21	n=33	n=39		n=72
Normal	30 (90.1%)	36 (92.3%)	0.748	66 (91.7%)
Erythema	1 (3%)	1		2
Impregnation	1 (3%)	2		3
Rash	1 (3%)	0		1
Day 28	n=17	n=26		n=43
Normal	16 (94.1%)	23 (88.5%)	0.116	39 (90.7%)
Erythema	1	1		2
Impregnation	0	2		2
Rash	0	0		0
Total	N=134 (person-time)	N=149 (person-time)		N=283 (person-time)
Normal	129 (96.3%)	132 (88.6%)	0.161	261 (92.2%)
Erythema	2	7		9
Impregnation	2	8		10
Rash	1	2		3

### Assessment of dressing use

Except for on Day 28, the study group had significantly lower pain scores than those of the control group (P<0.05), especially on Day 7 and Day 14 (P<0.01) (Table-V). From Day 7 to Day 28, the study group was significantly less prone to burning sensation than the control group (P<0.05), but both both groups felt anxious during dressing change (P>0.05). Dressing of the study group was changed significantly more easily (P<0.05), but the fixing outcomes were similar (P>0.05).

**Table-V:** Assessment of dressing use.

Wound-surrounding skin	Study group	Control group	P
Before wound treatment			
Pain	32.9±18.3	35.1±20.2	
Day 7			
Pain	17.5±7.2	33.1±15.5	<0.01
Burning sensation	3.4±0.8	2.5±1.4	<0.05
Anxiety	3.1±1.0	3.2±0.9	
Difficulty of changing dressing	3.1±0.8	2.3±1.4	<0.05
Fixing outcome	2.8±0.9	2.6±1.1	
Day 14			
Pain	3.4±1.8	17.3±8.2	<0.01
Burning sensation	3.9±1.2	2.7±1.6	<0.05
Anxiety	3.3±1.1	3.3±1.1	
Difficulty of changing dressing	3.5±0.9	2.6±1.3	<0.05
Fixing outcome	2.9±1.2	2.7±1.6	
Day 21			
Pain	1.1±0.9	8.6±5.7	<0.05
Burning sensation	3.7±0.9	2.9±1.3	<0.05
Anxiety	2.6±1.2	2.4±1.3	
Difficulty of changing dressing	3.8±0.8	3.1±1.1	<0.05
Fixing outcome	3.1±0.9	2.7±1.3	
Day 28			
Pain	2.7±1.5	3.4±2.1	
Burning sensation	3.8±1.1	3.0±1.4	<0.05
Anxiety	3.0±0.8	3.1±1.0	
Difficulty of changing dressing	3.7±1.0	2.4±1.0	<0.05
Fixing outcome	2.9±0.7	2.7±1.3	

P values are not given when there are no significant differences.

## DISCUSSION

Second-degree burn, which is the most common form of thermal burns, is difficulty to control owing to intense pain, slow healing and tendency to infections. Upon aggravation, it may form scars and ulcers which result in disfigurement or partial loss of function, thereby seriously affecting the quality of life.

Burn wounds are generally treated by changing dressing or surgery. Despite satisfactory wound healing outcomes, surgery is not suitable for all patients because of high cost. Dressing changing, on the other hand, is cheaper and mainly used now in clinical practice, but wounds heal slowly. Accordingly, it is of great significance to select proper dressings in order to control local infections and to promote healing. Ideal dressings should be easily applicable, well-penetrating and broad-spectrum, without drug resistance, local stimulation or systemic adverse reactions.^2^,^12^-^14^

Silver sulfadiazine cream has been used as wound dressing for over three decades,^10^,^15^ and in clinical application, 1% such cream can well control infections. However, it has failed in some cases due to enhanced drug resistance of bacteria. Meanwhile, this cream may inhibit leukocyte growth, easily induce allergic reaction, aggravate pain, and even hinder wound healing.

Recently, silver dressings have been used instead to resist pain and to accelerate wound healing following the mechanisms below.^16^-^18^ 1) Silver *per se*, as a metal, can hardly be absorbed by bacteria, but it can be ionized into the active form by wound secretions to bind cell membrane and proteins; 2) By interacting with sulfhydryl groups in the respiratory enzymes of microorganisms such as bacteria, silver is able to undermine the functions of these enzymes and some DNAs; 3) Silver preferentially inhibits the replication of bacterial DNAs by binding their bases. As a common, typical silver dressing, silver foam dressing is superior to other types owing to the better handling of exudates, air permeability, ease of tailoring and changing, as well as absence of residue.

In this study, second-degree burn patients were observed for 28 days and the effects of silver-containing soft-silicone foam dressing and silver sulfadiazine cream on non-joint positions were compared from the aspects of recovery, wound healing, detection rate of bacteria in secretions and assessment from patients and clinical practitioners. The recovery rates of the two groups were similar (P>0.05), but unrecovered patients in the study group had significantly higher proportions of repaired wounds (88%, 90% and 91% vs. 65% and 78%) (P<0.05). On Day 21, nine patients in the study group and three in the control group recovered, which became 25 and 16 respectively on Day 28, indicating that silver-containing soft-silicone foam dressing helped heal wounds more rapidly and effectively than the other one did. The study group had significantly higher proportions of repaired wounds from Day 7 to Day 21 (P<0.05), but the difference became insignificant with elapsed time to Day 28, suggesting that silver foam dressing worked more rapidly.

The bacterial culture-positive (exceeding 10^5^ organisms per gram of tissue) rates of the two groups were 54.8% and 50% respectively initially, which significantly reduced after treatment (*P*<0.01), and the rate of the study group was significantly lower on Day 28 (5.9% vs. 15.4%). Therefore, both types of dressings had high antibacterial activities, and the positive rates dropped on Day 7 for the study group and on Day 14 for the control group. Meanwhile, silver foam dressing was superior to silver sulfadiazine in the antibacterial performance and duration.

The study and control groups were observed 134 and 149 person-times respectively, with the normal wound-surrounding skin rates of 96.3% (129/134) and 88.6% (132/149) (P>0.05 except for on Day 14). Thus, silver foam dressing was safer than silver sulfadiazine.

Moreover, degrees of pain, burning sensation and anxiety were inquired upon dressing change. Except for on Day 28, the study group had significantly lower pain scores than those of the control group (P<0.05), especially on Day 7 and Day 14 (P<0.01). From Day 7 to Day 28, the study group was significantly less prone to burning sensation than the control group (P<0.05), but both both groups felt anxious during dressing change (P>0.05). The results suggested that silver foam dressing significantly alleviated pain more quickly than silver sulfadiazine did.

Dressing of the study group was changed significantly more easily (P<0.05), but the fixing outcomes were similar (P>0.05), indicating that it was more facile to use silver foam dressing. Silver foam dressing rapidly, readily and safely resisted wound bacteria, facilitated wound healing and shortened recovery time, effectively mitigating the pain of patients simultaneously.
